# Relationship between electronically monitored adherence to direct oral anticoagulants and ischemic or hemorrhagic events after an initial ischemic stroke—A case control study

**DOI:** 10.1371/journal.pone.0301421

**Published:** 2024-04-25

**Authors:** Katharina Rekk, Isabelle Arnet, Fine Dietrich, Alexandros A. Polymeris, Philippe A. Lyrer, Stefan T. Engelter, Sabine Schaedelin, Samuel S. Allemann

**Affiliations:** 1 Department of Pharmaceutical Sciences, Pharmaceutical Care Research Group, University of Basel, Basel, Switzerland; 2 Department of Neurology and Stroke Centre, University Hospital Basel and University of Basel, Basel, Switzerland; 3 Department of Geriatric Medicine, Neurology and Neurorehabilitation, Felix Platter Hospital, University of Basel, Basel, Switzerland; 4 Department of Clinical Research, University Hospital Basel and University of Basel, Basel, Switzerland; Sapienza University of Rome: Universita degli Studi di Roma La Sapienza, ITALY

## Abstract

**Background:**

Patients with atrial fibrillation (AF) have a high risk for recurrent clinical events after an ischemic stroke. Direct oral anticoagulants (DOAC) are prescribed for secondary prevention. Adherence to DOAC is crucial mainly because of their short elimination half-life. Non-adherence to DOAC can negatively impact patients’ outcomes. The relationship between (non-)adherence and recurrent clinical events is unknown in AF patients after initial stroke. We investigated adherence to DOAC in stroke survivors with AF who were included in the MAAESTRO study at the University Hospital Basel, Switzerland, between 2008 and 2022.

**Methods:**

This study is a secondary analysis of data from MAAESTRO with a matched nested case-control design and 1:2 ratio. DOAC intake was measured with a small electronic device (Time4Med^TM^). We defined two arbitrary intervals of 17 days and 95 days as the longest time spans with electronic monitoring data per patient to maximize the number of participants with adequate amount of observation time available for analysis. Taking and timing adherence were calculated retrospectively i.e., prior to the recurrent event for cases. Trendline analysis of adherence over 95 days was calculated. Linear regression analysis was performed after adjusting for the co-variables age and daily pill burden. Sensitivity analysis was performed with controls for intervals in the reverse direction (prospectively).

**Results:**

We analyzed 11 cases and 22 matched controls (mean age: 75.9 ± 9.2 years vs. 73.1 ± 8.4 years; n.s.) with similar stroke characteristics (NIHSS, mRS, MoCA) and 36.4% women in each group. Mean adherence values were high and similar between cases and controls (95 days taking: 87.0 ± 18.9% (cases) vs. 90.8 ± 9.8% (controls), n.s.; similar values for timing adherence). Six hemorrhagic and five ischemic events had occurred. Compared to controls, a significantly higher 95 days taking adherence was observed for hemorrhagic events (96.0 ± 5.0% (cases) vs. 88.1 ± 11.5% (controls); p<0.01) and a significantly lower 95 days taking adherence was observed for ischemic events (75.7 ± 24.8% (cases) vs. 94.2 ± 6.2% (controls), p = 0.024). Values for timing adherence were similar. A non-significant downward linear trend of adherence was observed over 95 days independently of the clinical events. The sensitivity analysis showed that the direction of the interval had negligible impact on the 95 days adherence.

**Conclusion:**

Because recurrent ischemic events after an AF-related stroke were associated with low adherence to DOAC <76%, adherence enhancing interventions seem crucial in anticoagulated AF-patients. However, AF-patients with high adherence might benefit from a regular re-assessment of the bleeding risk as hemorrhagic complications were associated with adherence to DOAC >96%.

**Trial registration:**

ClinicalTrials.gov NCT03344146.

## Introduction

Stroke is the second leading cause of death worldwide and the third leading cause of disability [1–3]. Stroke prevalence reaches 7% in the elderly [4] and is sex specific due to societal and biological gender-related factors [5]. Atrial fibrillation (AF) increases the risk of stroke. In patients with AF, the CHA_2_DS_2-_VASc score is used for risk stratification of ischemic stroke and thromboembolism [6, 7], while the HAS-BLED score indicates the risk of major hemorrhagic event [8].

Oral therapy with direct oral anticoagulants (DOAC) is essential for the secondary prevention of stroke in AF-patients [9]. Because of their short elimination half-lives and the lack of standard monitoring methods [9, 10], DOAC require a high level of adherence, that is, patients should take medications very thoroughly according to the recommendation of their health care providers [11]. There are different manners to measure adherence that generate data of various quality [12]. The electronic monitoring of medication intakes delivers objective data that enable to calculate specific adherence metrics [12, 13]. With electronic records, adherence can be estimated by taking the correct amount of the prescribed medicine (taking adherence) and at the appropriate time (timing adherence) [11].

Prevalence of recurrent events after an initial stroke is estimated at 1% after 30 days and raises to 19% within the first five days [14–17]. Reasons for recurrence are various and mostly non-modifiable such as age, previous stroke, congestive heart failure, among others [18]. However, non-adherence to anticoagulation therapy has been mentioned by researchers [19] and was even associated with negative clinical outcomes such as recurrent thromboembolic events [20]. Only few studies investigated the extent of non-adherence to anticoagulation therapy that leads to recurrent events after an initial stroke in AF-patients. A Spanish study included 132 AF-patients with electronic monitoring of the DOAC intake over 4 months [21]. Taking adherence was very high with a mean of 95.8% and thromboembolic events were more often observed in patients who failed to take >5% of the DOAC doses compared to patients who missed intakes only between 0 and 5% [21].

The MAAESTRO study was conducted at the Stroke Unit of the University Hospital Basel, Switzerland [22] (trial registration: ClinicalTrials.gov NCT03344146). Recruitment period started 01 January 2018 and ended 08 September 2021. All participants provided written informed consent. The MAAESTRO study aimed at evaluating the effect of an intake reminder on the adherence to DOAC in stroke patients with AF [23]. In brief, 130 stroke survivors were included who self-administered a DOAC after discharge [22]. In addition, patients recorded the DOAC intake with a small electronic monitoring device (Time4Med ^TM^ Adherence Innovations, Hong Kong, China) [13]. The study consisted of an observational phase of 3–6 months to collect baseline adherence data, followed by randomization to a group with reminder and a group without reminder for 3 months, and crossover for 3 more months [22]. During the 12-month MAAESTRO study, a total of 21 recurrent ischemic or bleeding events were observed in 16 patients (12.3%). These patients are the subject of the present study.

## Aims

To investigate the relationship between adherence to DOAC and clinical events in secondary stroke prevention in patients with AF. The hypothesis is that patients with a recurrent ischemic/hemorrhagic event will show a lower/higher level of adherence to DOAC in secondary prevention compared to patients with no recurrent clinical event.

## Materials and methods

### Study design and original data access

This study is a secondary analysis of data from the MAAESTRO study that was approved by the Ethics Committee of Northwest/Central Switzerland (EKNZ 2017–01552) and registered at ClinicalTrials.gov (NCT03344146). All participants gave written informed consent; study participation was voluntary. Original data were accessed on 08 January 2023. Authors had no access to information that could identify individual participants during or after data collection. We used a matched nested case-control design [24] that allows to study time-dependent exposures on rare outcomes where the use of randomized controlled trials (RCT) would be unethical [24]. This design allowed us to match a patient with a recurrent clinical event at a given point in time (case) to a patient with similar characteristics who had not experienced a recurrent clinical event (control). We used the STROBE case-control reporting guidelines [25].

### Case definition and cohort time axis

A patient was defined as case if they had developed a clinical event during the MAAESTRO study. A clinical event was defined as ischemic stroke, myocardial infarction, transient ischemic attack (TIA), intracranial hemorrhage, major extracranial hemorrhage or death [22]. For each case, we considered only the first recurrent event; subsequent recurrent events were not considered. In addition, adherence data had to be available prior to the clinical event. Patients with no adherence data were excluded.

### Selection of matching factors

Patient-related matching factors were sex, age, CHA_2_DS_2-_VASc score and HAS-BLED score as they are associated with a risk for clinical events. Allocation to MAAESTRO study phase (observational phase; interventional phase with or without reminder use) was incorporated as matching factor imposed by the RCT design of the MAAESTRO-study.

### Selection and number of controls

Patients who did not develop a recurrent event (i.e., controls) were randomly selected from the list of MAAESTRO patients who finished the study. Sampling was performed without replacement and with excluding the patient from the set. Once the list of all candidate controls was obtained, the final controls were selected with a randomly generated list of numbers. We selected a case-to-control ratio of 1:2 because it provides gains in statistical power [26].

### Stroke characteristics

Stroke severity was assessed on the **NIHSS** (National Institute of Health Stroke Scale; minor stroke, 1–4; moderate stroke, 5–15; moderate to severe stroke, 16–20; severe stroke, 21–24 [27]). Post-stroke functional status was assessed with the **mRS** (modified Ranking Scale; mild disability, 1; moderate disability, 2–4; extreme disability, 5 [28]). Cognitive screening was done with the **MoCA** (Montreal Cognitive Assessment; mild impairment, <26; severe impairment, <19 [29, 30]).

### Electronic monitoring data and adherence metrics

The electronic monitoring data were processed as described previously [31]. There were no missing data as lack of electronic data was defined as missed intake. Days of hospitalization, planned treatment pauses, and device dysfunctions were excluded from the calculation [31]. We selected two interval lengths of 17 days and 95 days to calculate adherence to DOAC. This arbitrary choice permitted to select the largest number of cases from the MAAESTRO study with the longest time span of electronic monitoring data available per patient, that is, 11 cases over 17 days and 9 cases over 95 days. We selected the interval prior to the recurrent event for cases, and prior to the last day of the MAAESTRO study i.e., observation or intervention phase for controls (i.e., retrospectively). We performed a sensitivity analysis and defined for controls the inverse direction of the interval (i.e., prospectively) that is, starting from the first day of the MAAESTRO observation or intervention phase.

Taking and timing adherence were calculated as percentage of the number of recorded doses divided by the number of prescribed doses during the predefined interval length. For timing adherence, we defined a grace period for the intake to occur within 25% of the median intake time i.e., ±3 hours for a twice daily regimen and ±6 hours for a once daily regimen [11, 22, 31].

### Statistical analysis

Microsoft^®^ Excel Version 16.54 (Microsoft Corporation, Redmond, WA, USA) was used for adherence calculations. SPSS statistics version 29 (IBM Corp., Armonk, New York, USA) was used for patient matching and statistical analysis. Numbers (with percentages) are presented for binary and categorical variables; means (± standard deviations, SD) or medians (with min-max) for continuous variables. Box-plots are presented with median (central line) separating the first and the third quartile; upper and lower whisker limits, and outliers (dots). Not normally distributed continuous variables (i.e., taking and timing adherence; characteristics of patients in the matched control groups) were evaluated using the Mann-Whitney U test. A p-value <0.05 was considered statistically significant with a two-sided approach for all patients and one-sided for specific events. Cases were subdivided into hemorrhagic and ischemic events and subgroup analysis was performed.

Weekly taking and timing adherence over the interval of 95 days were calculated for all patients. Trend-lines with simple linear regression (Y = aX+b) were calculated, with a negative slope (a) indicating downward trend of adherence over time. R-squared was calculated and averaged for cases and controls separately. Means with SD of the slope values were calculated for cases and controls separately. Linear regression analysis was performed after adjusting for the co-variables age and daily pill burden. We did not control for additional confounders because of much smaller groups than in the overall study. Retrospective data over the 95 days interval were used to calculate taking and timing adherence. Each model was fitted once in all pairs and once stratified by type of event.

During the medical visits that took place after the observation phase and after the intervention phase of the MAAESTRO study, patients were given the possibility to comment on their past intakes with a graphical visualization of the electronic monitoring data. Field notes were recorded by the investigator during the medical visits. Patients’ statements about difficulties with the DOAC intake are reported.

## Results

From the 16 eligible patients from the MAAESTRO study, five were excluded from the analysis because of missing adherence data following discharge (2), death (2) and technical issues (1). From the remaining 11 cases, six had developed hemorrhagic events (4 with major extracranial hemorrhage; 2 with intracranial hemorrhage) and five had developed ischemic events (4 with recurrent stroke or TIA; 1 with myocardial infarction). Five cases were observed during the observational phase (45.5%) and six during the interventional phase, of whom two used a reminder (18.2%) and four used no reminder (36.4%). The 11 cases were matched with 22 controls.

### Cohort characteristics

Mean age, sex distribution, mean risk scores and lifestyle factors were similar between cases and controls (Table 1). Stroke characteristics (NIHSS, mRS, MoCA) were comparable between cases and controls. All patients had at least two other medicines. Cases had more often hyperlipidemia compared to controls (100% vs 63.5%; p = 0.021). The use of anticoagulants (i.e., DOAC or vitamin K antagonists) prior to the initial stroke event was more often observed in cases compared to controls (72.7% vs 36.4%; p = 0.051). All patients were discharged with a DOAC, apparently with no preferred agent (Table 1).

**Table 1:** Baseline characteristics of 11 cases and 22 matched-controls issued from the MAAESTRO study. Statistically significant values are highlighted in bold.

	Cases			Control (n = 22)	p-value (2-sided)
	with hemorrhagic event (n = 6)	with ischemic event (n = 5)	All (n = 11)		
**Demographic data**					
• Age, mean ± SD [years]	78.0 ± 10.6	73.4 ± 7.5	75.9 ± 9.2	73.1 ± 8.4	0.379
• Male, n (%)	4 (66.7)	3 (60)	7 (63.3)	14 (63.6)	1
• CHA_2_DS_2-_VASc score, mean ± SD	6.0 ± 1.9	5.2 ± 1.3	5.6 ± 1.6	5.0 ± 1.4	0.220
• HAS-BLED score, mean ± SD	3.2 ± 0.8	3.2 ± 1.1	3.2 ± 0.9	3.0 ± 0.6	0.377
**Lifestyle factors**					
• Standard drinks per week, mean ± SD	5.7 ± 11.0	6.0 ± 8.7	5.8 ± 9.5	3.0 ± 3.1	0.202
• Smoking status, n (%)					0.304
○ No smoking	3 (50)	0 (0)	3 (27.3)	11.0 (50)	
○ Past smoking	2 (33.3)	4 (80)	6 (54.5)	8 (36.4)	
○ Ongoing smoking	1 (16.7)	1 (20)	2 (18.2)	2 (9.1)	
○ n.a.	0 (0)	0 (0)	0 (0)	1 (4.5)	
• Living alone, n (%)	1 (16.7)	2 (40)	3 (27.3)	9 (40.9)	0.458
• Employment status, n (%)					n.a.
○ Retired	5 (83.3)	4 (80)	9 (81.1)	9 (40.9)	
○ Employed	1 (16.7)	0 (0)	1 (9.1)	2 (9.1)	
○ n.a.	0 (0)	1 (20)	1 (9.1)	11 (50)	
• Education > 12 years, n (%)	3 (50)	0 (0)	3 (27.3)	13 (59.1)	0.090
**Comorbidities, n (%)**					
• Hypertension	5 (83.3)	5 (100)	10 (90.9)	19 (86.4)	0.717
• Hyperlipidemia	6 (100)	5 (100)	11 (100)	14 (63.6)	**0.021**
• Diabetes	2 (33.3)	1 (20)	3 (27.3)	4 (18.2)	0.562
• AF newly diagnosed during hospitalization, n (%)	1 (16.7)	2 (40)	3 (27.3)	6 (27.3)	1
**Medication**					
• Anticoagulation before initial stroke, n (%)	5 (83.3)	3 (60)	8 (72.7)	8 (36.4)	0.051
• DOAC at discharge, n (%)					0.174
○ apixaban	1 (16.7)	5 (100)	6 (54.5)	7 (31.8)	
○ rivaroxaban	2 (33.3)	0 (0)	2 (18.2)	4 (18.2)	
○ edoxaban	3 (50.0)	0 (0)	3 (27.3)	3 (13.6)	
○ dabigatran	0 (0)	0 (0)	0 (0)	8 (36.4)	
• DOAC switch or initiation after initial stroke, n (%)					**0.002**
○ switch	2 (33.3)	2 (40)	4 (36.4)	4 (18.2)	
○ new DOAC initiation	1 (16.7)	2 (40)	3 (27.3)	17 (77.3)	
○ no switch	3 (50)	1 (20)	4 (36.4)	1 (4.5)	
• DOAC Regimen, n (%)					0.458
○ QD	5 (83.3)	0 (0)	5 (45.5)	7 (31.8)	
○ BID	1 (16.7)	5 (100)	6 (54.5)	15 (68.2)	
• Daily pill burden, mean ± SD	7.8 ± 3.7	10.2 ± 3.6	8.9 ± 3.7	7.4 ± 3.3	0.246
• Later DOAC switch without reason, n (%)	0 (0)	1 (20)	1 (9.1)	1 (4.5)	0.619
**Stroke characteristics**					
• NIHSS at study start, mean ±SD	2.7 ± 4.7	1.4 ± 0.9	2.1 ± 3.4	1.5 ± 1.7	0.505
• mRS at study start, mean ±SD	1.0 ± 0.9	1.2 ± 0.8	1.1 ± 0.8	1.8 ± 0.9	**0.039**
• MoCA at study start, mean ± SD	23.7 ± 5.2	24.6 ± 2.1	24.1 ± 3.9	24.6 ± 4.9	0.769
• Neurorehabilitation after initial stroke, n (%)	1 (16.7)	1 (20)	2 (18.2)	11 (50)	0.082
• Pillbox use after initial stroke, n (%)	3 (50)	3 (60)	6 (54.5)	8 (36.4)	0.334

Regarding the anticoagulant agents used at the moment of the recurrent event, one switch from apixaban to rivaroxaban had occurred after hospital discharge without known reasons. From the 11 cases, five had a recurrent event under apixaban (45.5%), and three each under edoxaban and rivaroxaban (27.3%). No recurrent event was observed with dabigatran.

The median onset time for a recurrent **ischemic** event was 155 days (range: 47–214 days) for recurrent stroke; the myocardial infarction occurred after 231 days. The median onset time for a recurrent **hemorrhagic** event was 110 days (range: 32–182 days) for major extracranial hemorrhage and 266 days (range: 265–266) for intracranial hemorrhage. For one patient, the DOAC was discontinued two days before a planned surgical procedure, in accordance with guidelines [32], and hemorrhagic complications occurred during surgery. A recurrent event occurred after the re-initiation of the DOAC.

### Adherence

**Fig 1:**
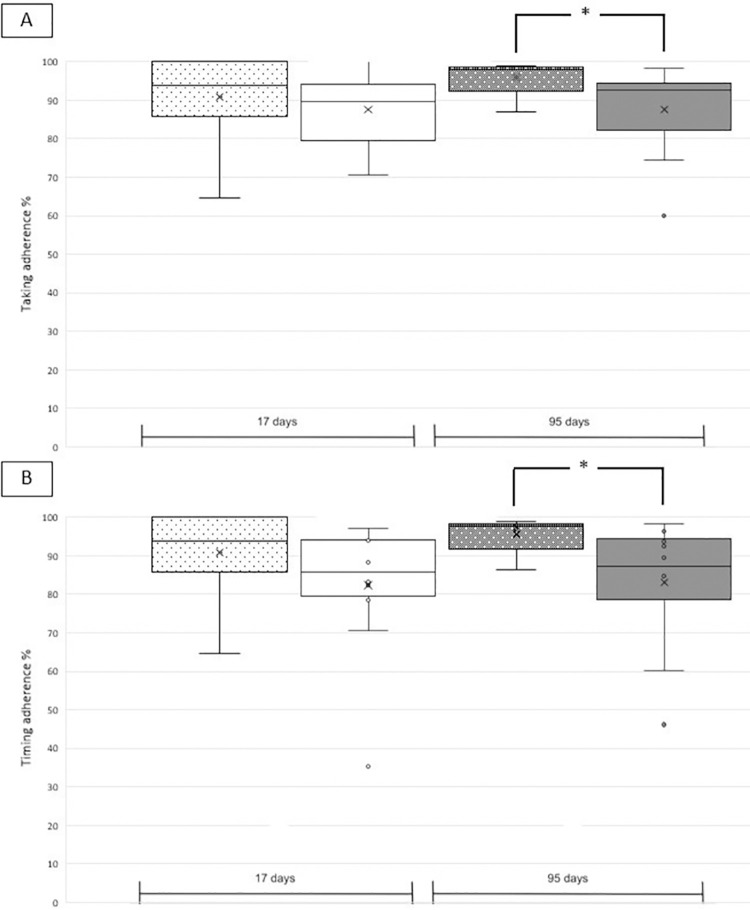
Box plots of six cases with hemorrhagic events (dotted) and 12 matched controls (plain) with taking adherence (upper panel A) and timing adherence (lower panel B) over the intervals of 17 days (white) and 95 days (grey). See Table 2, middle panel for the detailed values. Statistical significance is marked with an asterisk.

**Fig 2:**
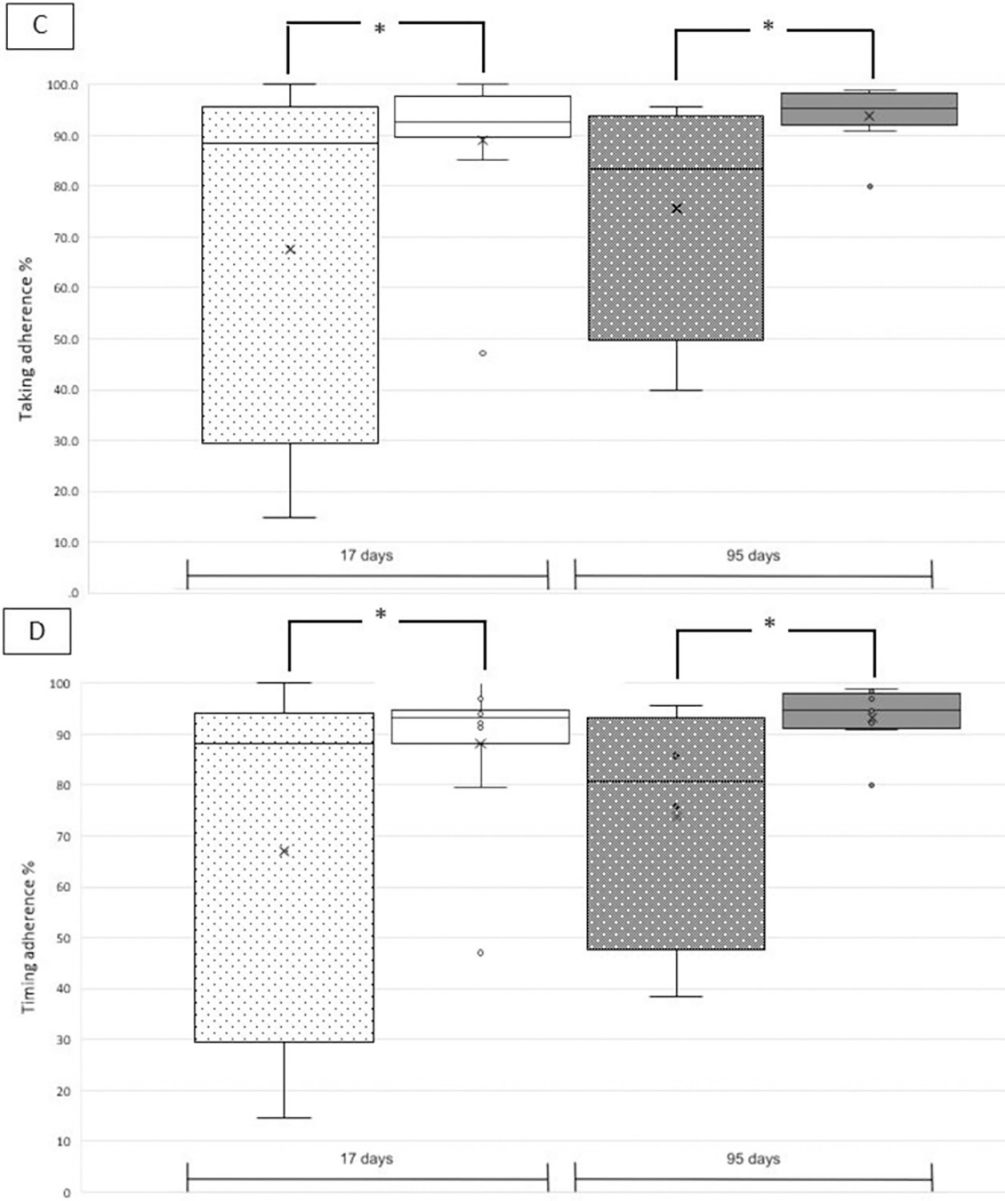
Box plots of five cases with **ischemic** events (dotted) and 10 matched controls (plain) with taking adherence (upper panel C) and timing adherence (lower panel D) over the intervals of 17 days (white) and 95 days (grey). See Table 2, lower panel for detailed values. Statistical significance is marked with an asterisk.

For two cases, the recurrent events occurred 17 days and 30 days after entry in the MAAESTRO study. These cases were excluded from the 95 days calculation because monitoring data were not enough. Taking and timing adherence values were similar for cases and controls over the interval of 17 and 95 days, respectively, and ranged from 80.1 ± 27.7% to 90.8 ± 9.8% (Table 2, upper panel). Differences were observed when ischemic and hemorrhagic events were analyzed separately. For **hemorrhagic** events, taking and timing adherence values over 95 days were significantly higher for cases compared to controls (taking adherence: 96.0 ± 5.0% vs 86.2 ± 11.1%; U = 6.0; p<0.01; Table 2, middle panel; Fig 1). For **ischemic** events, taking and timing adherence values were significantly lower for cases compared to controls independently of the interval length with values ranging between 67.1 ± 36.3% for cases and 95.8 ± 3.5% for controls (U = 40.5; p<0.05; Table 2, lower panel; Fig 2).

**Table 2:** Mean adherence values over the intervals of 17 and 95 days for all patients (11 cases and 22 controls for 17 days; 9 cases and 18 controls for 95 days; upper panel), and separately for hemorrhagic events (6 cases for 17 days and 5 cases for 95 days; middle panel) and ischemic events (5 cases for 17 days; 4 cases for 95 days, lower panel). Statistically significant values are highlighted in bold.

	Interval of 17 days				Interval of 95 days			
	Case (n = 11)	Control (n = 22)	p-value (2-sided)	U value	Case (n = 9)	Control (n = 18)	p-value (2-sided)	U value
**Taking adherence mean ± SD [%]**	80.4 ± 27.8	88.5 ± 20.0	0.281	150.0	87.0 ± 18.9	90.8 ± 9.8	0.781	75.0
**Timing adherence mean ± SD [%]**	80.1 ± 27.7	85.8 ± 23.0	0.585	135.5	86.0 ± 19.5	89.4 ± 9.7	0.668	72.0
	**Hemorrhagic events**							
	**Case (n = 6)**	**Control (n = 12)**	**p-value (1-sided)**	**U value**	**Case (n = 5)**	**Control (n = 10)**	**p-value (1-sided)**	**U value**
**Taking adherence mean ± SD [%]**	90.9 ± 13.2	82.4 ± 25.8	0.482	35.5	96.0 ± 5.0	88.1 ± 11.5	**0.010**	6.0
**Timing adherence mean ± SD [%]**	90.9 ± 13.2	77.6 ± 28.9	0.219	27.0	95.6 ± 5.1	86.2 ± 11.1	**0.007**	5.0
	**Ischemic events**							
	**Case (n = 5)**	**Control (n = 10)**	**p-value (1-sided)**	**U value**	**Case (n = 4)**	**Control (n = 8)**	**p-value (1-sided)**	**U value**
**Taking adherence mean ± SD [%]**	67.7 ± 36.7	95.8 ± 3.5	**0.028**	40.5	75.7 ± 24.8	94.2 ± 6.2	**0.024**	28.0
**Timing adherence mean ± SD [%]**	67.1 ± 36.3	95.5 ± 3.7	**0.020**	41.5	73.9 ± 25.0	93.6 ± 6.1	**0.024**	28.0

The linear regression analysis showed that neither age nor pill burden had significant effects on taking and timing adherence of controls compared to cases after adjusting for the co-variables. The difference in adherence between cases and controls was similar with approximately +15%-point difference for ischemic events and approximately -10%-point difference for hemorrhagic events (Tables 2 and 3).

**Table 3:** Results of the linear regression with the effect of group [control vs case] on taking adherence (left) and timing adherence (right) adjusted for age (upper panel) and daily pill burden (lower panel), stratified by type of event [ischemic, hemorrhagic]. Models are separated by a horizontal line.

		Taking adherence			Timing adherence			
Patients	Variable	Estimate	CI	p	Estimate	CI	p	N
**ischemic**	(intercept)	50.16	[-108.44; 208.76]		53.5	[-106.30; 213.29]		12
**event**	control (vs case)	19.94	[-2.97; 42.84]	0.08	20.92	[-2.15; 44.00]	0.07	
** **	age (in years)	0.34	[-1.74; 2.42]	0.72	0.27	[-1.83; 2.37]	0.78	
**hemorrhagic**	(intercept)	81.29	[30.75; 131.84]		89.28	[39.76; 138.80]		15
**event**	control (vs case)	-7.02	[-19.59; 5.55]	0.2472	-9.05	[-21.37; 3.26]	0.135	
** **	age (in years)	0.19	[-0.44; 0.82]	0.5293	0.08	[-0.54; 0.70]	0.782	
**ischemic**	(intercept)	88.36	[46.37; 130.36]		88.41	[46.59; 130.23]		12
**event**	control (vs case)	13.27	[-12.78; 39.31]	0.279	13.8	[-12.13; 39.73]	0.26	
** **	number of drugs	-1.27	[-5.12; 2.58]	0.474	-1.45	[-5.28; 2.38]	0.41	
**hemorrhagic**	(intercept)	97.85	[81.30; 114.40]		97.8	[81.84; 113.76]		15
**event**	control (vs case)	-7.89	[-20.23; 4.45]	0.19	-9.38	[-21.28; 2.52]	0.11	
** **	number of drugs	-0.22	[-1.78; 1.35]	0.77	-0.26	[-1.77; 1.25]	0.71	

### Adherence change prior to the recurrent event

**Fig 3:**
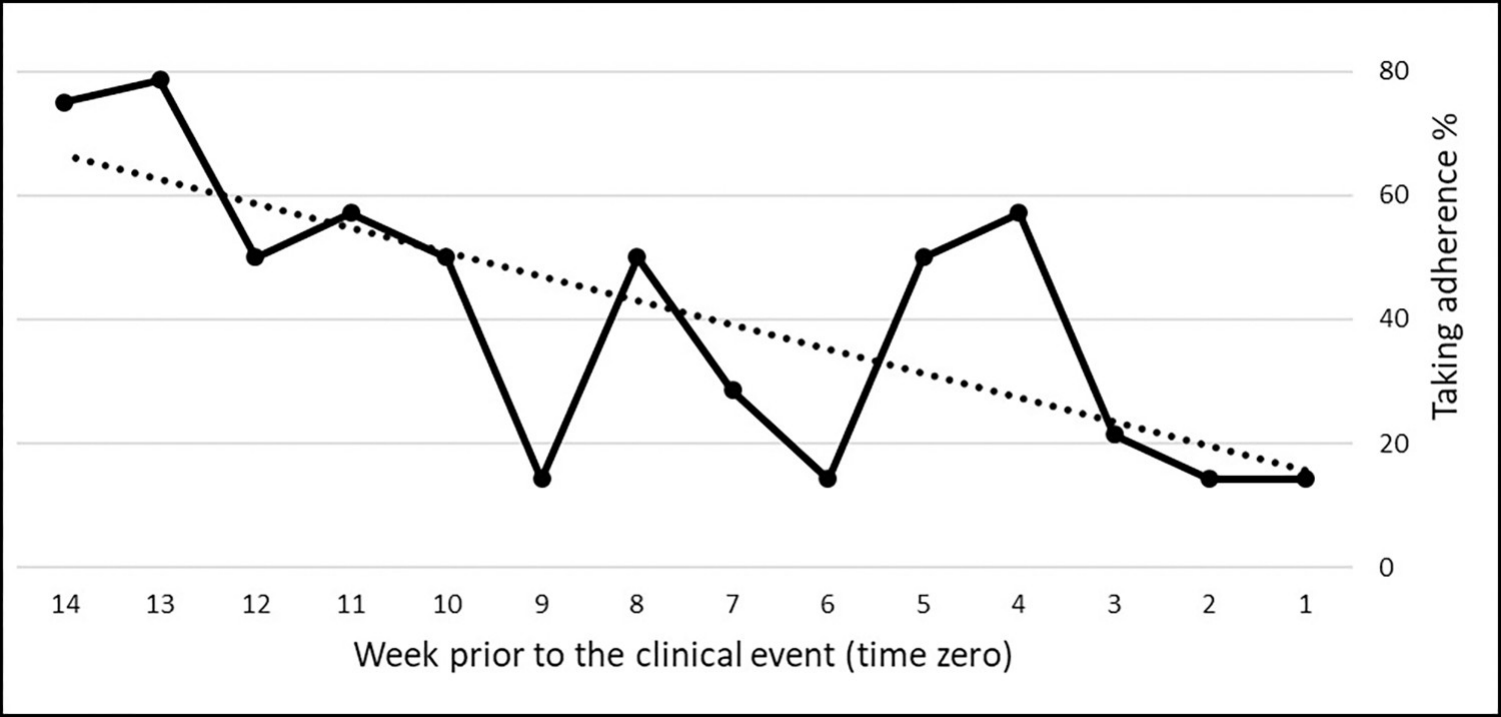
Illustrative example of a case under apixaban 5 mg twice daily with an overall taking adherence of 40% over 95 days prior to a myocardial infarction (time zero), with weekly mean taking adherence (bold line) showing a negative trendline (dotted line) with a slope of -3.9 and R^2^ of 0.509.

For 9 cases and 18 controls, mean weekly adherence (taking and timing) over 95 days prior to the recurrent event ranged from 100% to 14.3% (see illustrative example Fig 3). A downward sloping trend was observed in 5 (55%) cases and 8 (44%) controls for taking adherence, and in 5 cases and 10 controls (55% each) for timing adherence. The average slopes of the trendlines were similar in cases and controls with more pronounced negative slopes in cases (taking adherence: -0.7 ± 1.6% vs -0.2 ± 1.9%, n.s.; timing adherence: -0.7 ± 1.5% vs -0.4 ± 2.2%, n.s.; Table 3). Values were similar for hemorrhagic and ischemic events separately (Table 4). Mean R-squared were similar for taking and timing adherence and varied between 0.1903 ± 0.17 for cases (range: 0.0099–0.5088) and 0.163 ± 0.17 (range: 0–0.5023) for controls.

**Table 4:** Trend of weekly taking and timing adherence over 95 days, presented as means of slope ± SD, for all patients (9 cases and 18 controls) and separately for hemorrhagic events (5 cases) and ischemic events (4 cases).

	Case (n = 9)	Control (n = 18)	p-value (2-sided)	U value
**Taking adherence**	-0.7 ± 1.6	-0.2 ± 1.9	0.433	97.0
**Timing adherence**	-0.7 ± 1.5	-0.4 ± 2.2	0.561	93.0
	**Hemorrhagic events**			
	**Case (n = 5)**	**Control (n = 10)**	**p-value (1-sided)**	**U value**
**Taking adherence**	-0.8 ± 1.3	-0.7 ± 2.1	0.220	32.0
**Timing adherence**	-0.8 ± 1.2	-1.1 ± 2.5	0.340	29.0
	**Ischemic events**			
	**Case (n = 4)**	**Control (n = 8)**	**p-value (1-sided)**	**U value**
**Taking adherence**	-0.7 ± 2.2	0.5 ± 1.5	0.5	16.0
**Timing adherence**	-0.7 ± 1.9	0.5 ± 1.5	0.5	17.0

### Sensitivity analysis

Mean time span of adherence monitoring for controls was 126 ± 37 days (range: 95–187 days). Adherence values of controls did not differ if the direction of the intervals was analog to the cases i.e., retrospective (starting the **last** day of the study phase) or inverse to the cases i.e., prospective (starting the **first** day of the study phase). When mean adherence values of cases were compared to controls, the results were identical independently if controls were taken prospectively (Table 5) or retrospectively (Table 2), with the exception of 95 days timing adherence of the ischemic events that lost significant difference with prospective controls (Table 5).

**Table 5:** Taking and timing adherence over the intervals of 17 and 95 days for cases and controls with a prospective direction of the intervals (i.e., inverse to the cases).

	Interval 17 days				Interval 95 days			
	**Case (n = 11)**	**Control prospective (n = 22)**	**p-value 2-sided)**	**U value**	**Case (n = 19)**	**Control prospective (n = 18)**	**p-value (2-sided)**	**U value**
**Taking adherence mean ± SD [%]**	80.4 ± 27.8	88.3 ± 12.1	0.925	118.0	87.0 ± 18.9	90.5 ± 9.7	0.820	76.0
**Timing adherence mean ± SD [%]**	80.1 ± 27.7	85.0 ± 16.0	0.807	128.0	86.0 ± 19.5	87.8 ± 13.7	0.705	73.0
	**Hemorrhagic events**							
	**Case (n = 6)**	**Control prospective (n = 12)**	**p-value (1-sided)**	**U value**	**Case (n = 5)**	**Control prospective (n = 10)**	**p-value (1-sided)**	**U value**
**Taking adherence mean ± SD [%]**	90.9 ± 13.2	87.5 ± 9.2	0.193	45.5	96.0 ± 5.0	87.8 ± 11.4	**0.028**	9.0
**Timing adherence mean ± SD [%]**	90.9 ± 13.2	82.4 ± 16.7	0.090	50.5	95.6 ± 5.1	83.4 ± 16.6	**0.020**	8.0
	**Ischemic events**							
	**Case (n = 5)**	**Control prospective (n = 10)**	**p-value (1-sided)**	**U value**	**Case (n = 4)**	**Control prospective (n = 8)**	**p-value (1-sided)**	**U value**
**Taking adherence mean ± SD [%]**	67.7 ± 36.7	89.2 ± 15.4	0.083	13.5	75.7 ± 24.8	93.9 ± 6.2	**0.037**	27.0
**Timing adherence mean ± SD [%]**	67.1 ± 36.3	88.1 ± 15.4	0.083	13.5	73.9 ± 25.0	93.3 ± 6.1	0.055	26.0

### Patients’ statements

Three cases reported difficulties with the DOAC use prior to the recurrent event. One patient was taking a twice daily medication only once a day without knowing his error prior to a recurrent myocardial infarction. One patient mentioned 1–2 missed doses per week; his taking and timing adherence were 93.8% over 17 days and 98.9% over 95 days prior to a recurrent intracranial hemorrhage. One patient reported psycho-social problems because of the death of a family member; her adherence was 100% 17 days prior to a major extracranial hemorrhage.

## Discussion

This study provides a detailed insight into the adherence of patients with AF-related ischemic stroke who developed a recurrent event despite being appropriately anticoagulated with DOAC. Adherence to DOAC over 95 days prior to the event was high with mean taking adherence values of 87.0 ± 18.9% for cases and 90.8 ± 9.8% for controls. These results appear to be in line with those from previous studies with electronic measure of adherence to DOAC. As an example, taking adherence of 90.8% and 89.2% at 6 and 12 months were observed after a mixed intervention [33, 34]. However, in our study, adherence prior to the recurrent event was significantly higher in patients with recurrent hemorrhagic events, and lower in patients with recurrent ischemic events compared to matched controls. In other terms, a mean taking adherence above 96% was associated with hemorrhagic events, while a value below 76% was associated with ischemic events. In addition, the weekly adherence showed a moderate downward sloping trend in patients with a recurrent event, albeit without statistical significance. To our knowledge, this is the first time that levels of non-adherence to DOAC are directly associated to recurrent events in stroke survivors with AF with a distinction between hemorrhagic and ischemic events.

### Study design

We selected a nested case-control design for several reasons. The case-control is an observational epidemiological method used to evaluate factors when the outcome is infrequent [35] or occurs after a long latent period [36]. Even if an initial stroke increases the risk for a second stroke [15, 17], recurrent events after a stroke while on appropriate anticoagulation are uncommon with an estimated prevalence of 4.6% within 3 months after the index stroke, calculated from 2,082 patients in 11 centers in Switzerland, Germany and the USA [19]. Incidences from population-based studies range from 5.1% in Korea [37] to 15.5% in Italy [38]. Consequently, we have extrapolated that between 6 and 20 patients with recurrent events would be best identified from the 130 patients recruited in the MAAESTRO study. Further, the nested design enables to perform a study within a cohort and compare patients who developed the event of interest with randomly selected patients among those who did not develop the event. One of the challenges in case-control studies is the selection of the matching factors to obtain a similar distribution of patients’ characteristics. The intervention in the MAAESTRO study was aimed at increasing adherence to DOAC and was divided in two different phases i.e., with and without reminder use. Previous findings showed that reminder use increases medication adherence [34]. Thus, the allocation to the phase of MAAESTRO (observational phase; interventional phase with or without reminder use) was incorporated as matching factor to reduce confounding effect. By doing so, we diminished the risk of selection bias and obtained a higher level of evidence. Finally, it seemed unethical to develop a randomized controlled study on (non-)adherence to secondary prevention medicine with the endpoint being a debilitating recurrent event such as a myocardial infarction or a severe hemorrhage.

### Recurrent events

Among the 130 patients who participated in the 12-month MAAESTRO study after an AF-related ischemic stroke, a total of 21 recurrent events were observed in 16 patients (12.3%). This is similar to the cumulative incidence of 8.3% AF-patients from 8 centers in the USA who developed ischemic events (6.7%) and intracranial hemorrhage (1.6%) within 90 days of a cardioembolic stroke [39]. In our cohort study, six cases (4.6%) developed hemorrhagic event, which are expected complications in DOAC-treated patients [40]. However, when compared to vitamin K antagonists, the risk of intracranial hemorrhage is reduced [41] and associated with better outcomes [40]. Further, when balancing between thromboembolic risk, clinical outcome, and hemorrhagic risk, the sentence “fear the clot not the bleed” [42] places the emphasis clearly on avoiding a recurring thromboembolic event. In our study, cases with hemorrhagic events had taking adherence values of 91% over 17 days and 96% over 95 days, which are much higher than the usual cutoff of 80% used in adherence studies [43]. The use of the 80% cutoff may explain why no difference for hemorrhagic events was observed between adherent or non-adherent patients in a systematic review [44]. Thus, interventions to optimize chronic DOAC treatment in AF-patients should not be limited to adherence enhancing actions in view of avoiding ischemic events. The regular re-assessment of bleeding risk might be equally important because hemorrhagic complications were associated with high adherence to DOAC >96%.

In our study, all cases and 64% of controls presented with dyslipidemia at study entry. This corresponds to a well-known risk factor for stroke, as a history of hyperlipidemia has been statistically associated with recurrent ischemic events [37, 39], among the complex interplay of stroke risk factors. Thus, our patients’ characteristics coincide with the usual stroke risk factors and our results are generalizable.

### Electronic monitoring

Among the different methods to measure adherence, we selected the electronic method because it is the gold standard in adherence research [45]. One persistent concern with any indirect method of adherence measure is that the recording does no guarantee the ingestion of the medicine. Moreover, a Hawthorne effect (i.e., an “aware or unconscious complex behavior change in a study environment” [46]) cannot be excluded when individuals know that they are observed [31, 47]. Although all medical research with observational design is prone to the Hawthorne effect, its significance disappears in well-designed RCT [46] because they are designed to reduce the risk of bias. Moreover, it can be assumed that all groups are equally expose to the Hawthorne effect so that its impact on the main outcome may be reduced [48]. Finally, in clinical studies where adherence measurement is unblinded and part of the intervention, it is unlikely that patients register the intakes without ingesting the medication.

### Interval lengths and direction

The length of 3 months is the most commonly used period of use for electronic bottles in adherence studies, with a range between one week to 24 months [49]. Reasons in favor of 3 months are that this length of time is needed to develop a reliable medication habit [50] and it gives sufficient information about the patient’s adherence [51]. For the data collection of the controls, we selected the same direction as for the cases, that is, retrospectively prior to the last day of the MAAESTRO study phase (observation or intervention phase). The sensitivity analysis showed that the reversing of the direction had a negligible effect on the adherence values, with a difference of approximately 2% for all patients and a partial loss of the significance only for the 95 days timing adherence of the ischemic events. Because of the low number of 4 cases for this analysis, this result must be interpreted with caution. Nevertheless, taking adherence values and the interval length of 95 days seem representative for long-term adherence calculation, and deliver robust results.

### Stroke characteristics

We used three different scores to capture the functional disabilities after an acute stroke, according to medical practice. The NIHSS estimates stroke severity, the mRS functional status, and MoCA cognitive impairment. In our study, all cases and controls had minor stroke (mean NIHSS <2.2) and low functional and cognitive deficits (mean mRS <2 and mean MoCA <25). This is likely to be the consequence of one specific inclusion criteria of the MAAESTRO study, namely that patients had to be able to manage their medication by themselves, which require cognitive and functional capacities. The severity of stroke, as measured by NIHSS, can influence the ability of patients to attain high levels of adherence to medication. Similarly, cognitive impairment, even mild, can contribute to poor adherence [52, 53]. Thus, it was not surprising to observe high adherence values in our cohort of patients who had little post-stroke impairment. Nevertheless, cases with a recurrent event had a significantly suboptimal adherence compared to controls, which emphasizes the deep individual nature of medication adherence. Finally, post-stroke impairment has been reported to affect multiple cognitive domains [54], of which some may be related to medication adherence.

### Trendline analysis of adherence

A slight downward sloping trend of the adherence prior to the recurrent event was observed. This trend fits common sense for ischemic events, but might be counterintuitive for hemorrhagic events in view of mean 95 days adherence values that were lower for the former and higher for the latter. In fact, the trendlines of the cases and controls were moderate and mean R-squared values were below 0.2, indicating that the association is weak and that a linear relationship between adherence fluctuation and time is unlikely. Furthermore, the illustrative case presented is exceptional with an overall taking adherence of 40% over 95 days, and a drop to 14.3% the week prior to the recurrent event, which equals to the intake of two tablets per week. More data are needed to investigate adherence trajectories over time.

### Strengths and limitations

We acknowledge several strengths. Firstly, we used a nested design and matched cases to controls from the same study population. Thus, we were able to identify infrequent outcomes (i.e., recurrent events in AF-related ischemic stroke patients) in a setting where randomization would be unethical. Secondly, we selected matching factors that are associated with the outcome of interest (i.e., recurrent event) and thus, are appropriate. Thus, overmatching was avoided and the statistical efficiency of the analysis was guaranteed [24]. Thirdly, we generated highly detailed information about adherence before a recurrent event through the use of electronic monitoring. This degree of insight and knowledge is unprecedented in the literature. Consequently, the association between the main results (i.e., adherence) and the outcome (i.e., clinical events) is of good evidence. Fourthly, the original MAAESTRO study has generated data with high quality that already permitted another secondary data analysis [55]. Fifthly, we used a small electronic device to monitor the medication intake, Time4Med^TM^, that was well accepted by the patients. This method delivers objective real-life data and gives credibility to the adherence metrics and thus, to our overall results.

We acknowledge some limitations. Firstly, our sample size with 11 survivors of AF-related ischemic stroke is relatively small. Thus, our results need to be interpreted with caution. Nevertheless, the methodological rigor of our design gives robustness to our results. To confirm our findings, a larger sample size might be necessary. Secondly, the results are limited to an elderly Swiss population from the University Hospital Basel, Switzerland. Thus, the generalizability of our findings is limited. For further analysis, the sample needs to be more heterogeneous.

## Conclusion

Our study showed that recurrent events occurred in AF-stroke patients under DOAC treatment, with ischemic events being associated with low adherence to DOAC and hemorrhagic complications being associated with high adherence to DOAC. Thus, even if adherence-enhancing interventions seem crucial in anticoagulated AF-patients, a regular re-assessment of the hemorrhagic risk might be equally appropriate. Thus, interventions to optimize chronic DOAC treatment in AF-patients should include a regular re-evaluation of the adherence and a re-assessment of the hemorrhagic risk. Our results might influence the counseling of DOAC patients after a stroke.

## Supporting information

S1 File Raw adherence values (taking and timing) with prospective or retrospective interval for cases and controls over 95 days and 17 days. (XLSX)

S2 File Raw variables including age and daily pill burden for 11 cases and 22 matched controls for the linear model calculation. (XLSX)

S3 File Raw weekly adherence values (taking and timing) for 9 cases and 18 matched controls including date of clinical event and start date of calculation. (XLSX)
